# A pulse of summer precipitation after the dry season triggers changes in ectomycorrhizal formation, diversity, and community composition in a Mediterranean forest in California, USA

**DOI:** 10.1007/s00572-018-0859-3

**Published:** 2018-08-13

**Authors:** Takeshi Taniguchi, Kuni Kitajima, Greg W. Douhan, Norikazu Yamanaka, Michael F. Allen

**Affiliations:** 1 Center for Conservation Biology, University of California, Riverside, CA 92521 USA; 2 Arid Land Research Center, Tottori University, Tottori, 680-0001 Japan; 3 Department of Plant Pathology and Microbiology, University of California, Riverside, CA 92521 USA; 4 University of California Cooperative Extension, 4437-B S. Laspina St., Tulare, CA 93274 USA; 5 Department of Biology, University of California, Riverside, CA 92521 USA

**Keywords:** Basidiomycota, Community structure, Drought, Precipitation, Pulse rain, Short-term change

## Abstract

Rapid responses of microbial biomass and community composition following a precipitation event have been reported for soil bacteria and fungi, but measurements characterizing ectomycorrhizal fungi remain limited. The response of ectomycorrhizal fungi after a precipitation event is crucial to understanding biogeochemical cycles and plant nutrition. Here, we examined changes in ectomycorrhizal formation, diversity, and community composition at the end of a summer drought and following precipitation events in a conifer–oak mixed forest under a semiarid, Mediterranean-type climate in CA, USA. To study the effects of different amounts of precipitation, a water addition treatment was also undertaken. Ectomycorrhizal fungal diversity and community composition changed within 6 days following precipitation, with increased simultaneous mortality and re-growth. Ectomycorrhizal diversity increased and community composition changed both in the natural rainfall (less than 10 mm) and water addition (50 mm) treatments, but larger decreases in ectomycorrhizal diversity were observed from 9 to 16 days after precipitation in the water addition treatment. The changes were primarily a shift in richness and abundance of Basidiomycota species, indicating higher drought sensitivity of Basidiomycota species compared with Ascomycota species. Our results indicate that ectomycorrhizal formation, diversity, and community composition rapidly respond to both precipitation events and to the amount of precipitation. These changes affect ecosystem functions, such as nutrient cycling, decomposition, and plant nutrient uptake, in semiarid regions.

**Electronic supplementary material:**

The online version of this article (10.1007/s00572-018-0859-3) contains supplementary material, which is available to authorized users.

## Introduction

Dryland ecosystems, defined as those having a precipitation: evapotranspiration ratio less than 1, occupy approximately 35–45% of the Earth’s land area (Asner et al. 2003; Reynolds et al. 2007; Jenerette et al. 2012). Rainfall in dryland ecosystems has seasonal patterns, but the actual amount and timing varies seasonally and annually and is highly unpredictable (Noy-Meir 1973). Water from episodic precipitation events can cause some organisms to undergo a pulse of a biological activity. Pulse responses of microorganisms and plants increase the biogeochemical fluxes of carbon and nitrogen (Austin et al. 2004; Huxman et al. 2004; Inglima et al. 2009; Yahdjian and Sala 2010; Dijkstra et al. 2012). Biological transformation of nitrogen by microorganisms is also stimulated by the water supply (Yahdjian and Sala 2010; Dijkstra et al. 2012).

Just as important, the degree of the response is affected by the amount of water supplied and by the delivery pattern (Sponseller 2007; Jenerette et al. 2008; Nielsen and Ball 2015). During the twenty-first century, precipitation regimes in dryland ecosystems are expected to change in response to global warming (Sala et al. 1992; Golluscio et al. 1998; Stocker et al. 2013). In areas with a Mediterranean climate, increases in mean temperature and decreases in annual rainfall have been reported (Solomon 2007). Changes in precipitation patterns and amounts also affect carbon cycling, especially in these regions (Rey et al. 2005; Inglima et al. 2009). At the James Reserve in southern CA, USA, a suite of downscaled global circulation models show increasing variation in precipitation, even as temperatures gradually climb (Allen et al. 2014). Therefore, knowledge of the effect of a pulse of rain on soil biota, which causes shifts in biogeochemical fluxes, is needed to predict the influence of climate change on dryland ecosystems.

Rapid responses to precipitation events have been reported for fungi and bacteria (Allen 1993; Landesman and Dighton 2011; Placella et al. 2012; Hernandez and Allen 2013; Allen and Kitajima 2013). Abundance of some bacteria increased within 1 h after a precipitation event (Placella et al. 2012), and an increase in fungal biomass was observed within 3 h (Landesman and Dighton 2011). Rapid responses to water supply by some microorganisms, such as ammonia-oxidizing bacteria, cyanobacteria in biocrusts, and arbuscular mycorrhizal fungi, have also been reported (Bowling et al. 2011; Placella and Firestone 2013; Yang et al. 2016). Ectomycorrhizal (ECM) fungi are a functional group that is most closely involved in carbon and nitrogen cycling, as well as plant nutrient uptake. However, knowledge on the short-term responses of ECM roots and community to precipitation is limited, and the results are equivocal (Querejeta et al. 2009; Hawkes et al. 2011).

ECM fungi are a keystone group of microorganisms in forest ecosystems, where they play important functional roles, notably by regulating biogeochemical cycles and improving plant nutrition (Smith and Read 2008). In a Californian Mediterranean-type forest, 27% of total net primary production was allocated to ECM fungi in 2011 (Allen and Kitajima 2014). ECM fungal biomass can comprise a substantial portion of the carbon pool, constituting up to 15% of soil organic matter in some ecosystems (Vogt et al. 1982). When ECM roots die, carbon is deposited as litter. ECM root tips are estimated to turnover at times ranging from 139 days to 5 years (Rygiewicz et al. 1997; Majdi et al. 2001; Treseder 2004). The decomposition rate also differs among ECM fungal species (Wilkinson et al. 2011; Fernandez and Koide 2012).

The number of ECM fungal species has been estimated to range from 5000 to 6000 (Molina et al. 1992) up to 20,000 to 25,000 (Rinaldi et al. 2008; Brundrett 2009). In addition to variations in their decomposition rates, different ECM fungal species vary in terms of their responses to environmental variables and their relationships with host plants (Cairney 1999; Taniguchi et al. 2007). Different ECM fungal species differentially alter the allocation of resources to their hosts (Dickie et al. 2002; Allen et al. 2003; van der Heijden and Kuyper 2003), and they degrade organic materials by producing extracellular enzymes (Pritsch et al. 2004; Courty et al. 2005; Taniguchi et al. 2008). Therefore, it is important to assess the response of the ECM fungal community to perturbations such as drought and precipitation, because changes in the ECM fungal community will affect the nutrient uptake and productivity of host plants.

Soil moisture is one of the factors that regulate ECM fungal colonization and fungal community composition (Swaty et al. 2004; Buée et al. 2005; di Pietro et al. 2007; Richard et al. 2011). Valdés et al. (2006) observed a reduction of ECM root biomass in a drought year, compared with a non-drought year. Swaty et al. (2004) reported that ECM colonization was lower in a dry site, with higher tree mortality, compared with a mesic site with low tree mortality. Furthermore, there was a shift in the ECM fungal community composition. In summer, *Cenococcum geophilum* was dominant, and this fungus remained metabolically active under a low soil water potential when other ECM fungi stopped functioning (Pigott 1982; Jany et al. 2003; Buée et al. 2005). The effect of soil moisture on the ECM fungal community has generally been examined at intervals in the order of months to years. Courty et al. (2008) examined the temporal change in the ECM fungal community in a temperate oak forest, and they observed that the abundance of some species exhibited seasonality. The turnover of the ECM fungal species composition occurred over the course of 1 month. However, the responses of fine roots and fungi may not occur over weeks to months, but over days (Stewart and Frank 2008; Allen and Kitajima 2013). Short-term responses (within 1 month) of ECM fungi to rainfall have not been reported on ECM roots but ECM hyphae (Allen and Kitajima 2013). In addition, the effect of the amount of rainfall on ECM fungal composition has not been examined.

Here, we examined the short-term responses of the colonization, diversity, and community composition of ECM roots during a monsoonal rain event in a conifer–oak mixed forest in a semiarid region of CA, USA, which has a Mediterranean climate. To assess the effects of different amounts of precipitation, a water addition (WA) treatment, in addition to a natural rainfall (NR) treatment, was also established. We hypothesized that ECM fungal biomass would increase, the community structure would change in response to precipitation, and the responses would differ with the amount of precipitation.

## Materials and methods

### Study site and experimental design

The study site was located in the James Mount San Jacinto Reserve (http://www.jamesreserve.edu), a University of California Natural Reserve (33° 48′ 30′′ N, 116° 46′ 40′′ W). The elevation is 1500 m, and precipitation occurs mostly as rain from November to April. The mean annual precipitation was 652 mm (1961–2010) (Allen and Kitajima 2014), but only 437 mm of precipitation fell in 2011, when this study was undertaken. Additional details of the climate have been presented previously (Allen 2007; Vargas and Allen 2008; Hernandez and Allen 2013; Kitajima et al. 2013; Allen and Kitajima 2014). The James Reserve is a mixed conifer and oak forest that is dominated by ECM plants such as black oak (*Quercus kelloggii* Newberry) and ponderosa pine (*Pinus ponderosa* C. Lawson) (Kitajima et al. 2013). As described by Vargas and Allen (2008), the James Reserve was a test site for the National Ecological Observatory Network, with an extensive wireless network of environmental sensors (Allen 2007; Rundel et al. 2009).

Within this site, one quadrat that includes a soil ecosystem observatory was established around each of three black oak trees. In each quadrat, the seasonal change in ECM fungal diversity was measured. The sizes of each quadrat were 2.4, 3.0, and 2.4 m^2^, respectively. The difference in the quadrat size was due to the size of a soil ecosystem observatory. Each quadrat was 4.0, 2.0, and 1.6 m from the nearest black oak trees and more than 13.0 m away from each other.

Additionally, two of the three black oak trees were selected to examine the effect of precipitation on short-term responses of the ECM fungal community. Six quadrats were established around the two black oak trees (in total, 12 quadrats) (Fig. S1). One of the six quadrats in each of the two black oak trees included a soil ecosystem observatory in it (Fig. S1). The other two soil ecosystem observatories were established outside the quadrat, but within 5 m from the two black oak trees to measure the soil environmental condition of the NR treatment. The sizes of quadrats with soil ecosystem observatories were 2.4 m^2^, and those without the observatories were 0.6 m^2^. The distance between two black oak trees was 32.0 m. Each quadrat was 1.0 to 4.0 m from the nearest black oak trees and 1.5 to 4.5 m away from each other (Fig. S1). Three quadrats were set as a NR treatment, and the other three were set as the WA treatment.

On 30 and 31 July 2011, after summer drought, there was a minor monsoonal rainstorm (see Fig. 3, Allen and Kitajima 2014). At the weather station in James Reserve, the total volume of recorded rainfall was less than 10 mm. Additionally, on 1 August, we added 50 mm of water to the WA treatment to simulate a major monsoonal rainstorm.

### Sensor data

Six soil ecosystem observatories were running as described above. One soil ecosystem observatory point, including one automated minirhizotron (RhizoSystems, LLC, Riverside, CA, USA), was located in the NR treatment and captured daily images. Images are available (http://ccb.ucr.edu/lab_protocols.html) and imagery analyses for this location are published (AMR8, Allen and Kitajima 2014). Details describing the sensor deployment, including temperature, soil water, and soil CO_2_, all at three depths (2, 8, and 16 cm) have been described by Allen and colleagues (Allen 2007; Rundel et al. 2009; Allen and Kitajima 2013; Hernandez and Allen 2013; Allen and Kitajima 2014). At each soil ecosystem observatory, three types of belowground sensors, which measured soil temperature (HOBO Weather Station 12-bit Temperature Smart Sensor; Onset, Cape Cod, MA, USA), soil water content (HOBO Weather Station Soil Moisture Smart Sensor), and CO_2_ concentration (Vaisalia, GMP222; Vaisalia, San Jose, CA, USA), were established. The three types of sensors were buried at three depths (2, 8, and 16 cm). Soil respiration was calculated using the flux-gradient method based on the CO_2_ concentrations in the soil profile (Tang et al. 2005; Vargas and Allen 2008).

### Sampling the soil and black oak roots

In the beginning of May, June, and July, a composite soil sample from eight soil core samples containing black oak roots was collected in three quadrats near soil ecosystem observatories at a depth of 0 to 20 cm with a soil core sampler (*ϕ* = 2.0 cm). Coincidently with a monsoon rainfall event on 30 and 31 July, soils containing the black oak roots were also collected on 1 August. Then, water was added to the WA treatment. On 3, 5, 8, and 15 August, a total of 12 composite soil samples were collected from a total of 12 quadrats on each date. To collect a composite soil sample, eight soil core subsamples were taken in each quadrat, and the soil subsamples were mixed. After returning to the laboratory, roots were sieved from the soil cores and washed within 2 weeks after sampling. The black oak roots were observed under a dissecting microscope. Microscope observation for each composite sample was done within 20 h after washing. They were defined as ECM roots if they lacked root hairs and were covered with a mantle, and they were defined as non-mycorrhizal (NM) roots if they had root hairs and lacked a mantle. The percentage of ECM roots was calculated as: (the number of ECM root tips × 100) / the total number of root tips. ECM root tips clipped were used for morphotyping and then DNA extraction.

### Classification and identification of ECM fungal species

All of the ECM root tips from each composited sample were classified into morphotypes, based on their surface color, texture, emanating hyphae, and rhizomorphs according to Agerer (1997). One to two ECM root tips from each morphotype of each sample was chosen and used for DNA analysis to identify the ECM fungal species (Gardes and Bruns 1993). Each ECM root tip was first washed by vortexing it three times in 100 μl of a cetyltrimethylammonium bromide (CTAB) solution to remove contamination.

The ECM roots were covered by Kimtowel, then dried, and crushed by hand. The ECM roots crushed were placed in tubes containing 10 μl of extraction buffer from the Extract-N-Amp PCR Kit (Sigma-Aldrich, St. Louis, MO, USA). To extract DNA, the tubes were treated by a 10 min at 60 °C incubation and a 10 min of thermal shock at 95 °C in a thermal cycler (MyCycler, Bio-Rad Laboratories Inc., Hercules, CA, USA), then 10 μl of dilution solution supplied with the Kit was added. The internal transcribed spacer region of ribosomal DNA was amplified with the fungal-specific primers ITS1F (Gardes and Bruns 1993) and ITS4 (White et al. 1990). A 1.0-μl aliquot of extracted DNA was combined with 6.25 μl of AmpliTaq Gold 360 Master Mix (Applied Biosystems, Foster City, CA, USA) in a 12.5-μl reaction. The thermal profile in the polymerase chain reaction (PCR) was as follows: a 10-min initial denaturation at 95 °C, followed by 35 cycles of denaturation for 30 s at 95 °C, annealing for 30 s at 55 °C, and extension for 1 min at 72 °C, followed by a 7-min final extension at 72 °C. Amplicons were visualized in 0.7% agarose gels that were stained with SYBR Green I (Molecular Probes, Eugene, OR, USA). If the DNA band was not observed or the PCR product had more than two bands, DNA was extracted in CTAB from another root tip of the same morphotype (Gardes and Bruns 1993), and then another PCR was run as described above. Amplicons were cleaned with exonuclease I and shrimp alkaline phosphatase (Glenn and Schable 2005). Sequencing was performed with primers ITS1F or ITS4 on an ABI PRISM 3700 genetic analyzer (Applied Biosystems) located at the Core Instrumentation Faculty of the University of California at Riverside’s Institute for Integrative Genome Biology. For the unsuccessful samples that had more than one fungal sequence, PCR products were purified with the QIAquick Gel Extraction Kit (Qiagen, Hilden, Germany), and then they were cloned with the TOPO-TA Kit (Invitrogen, Carlsbad, CA, USA) and propagated in *Escherichia coli*. A successful clone from each PCR product was grown overnight in Luria–Bertani medium containing 100-μg/ml ampicillin. Plasmid DNA was purified from the cultures with the FastGene Plasmid Mini Kit (Nippon Genetics Co., Ltd., Tokyo, Japan), and the total of successful 45 clones were sequenced with primer M13F.

Sequences were edited using MEGA 5.0, before being preliminarily identified to the family or order level using nucleotide Basic Local Alignment Search Tool searches of GenBank (http://www.ncbi.nlm.nih.gov/blast). Sequences were aligned for each family or order, and then they were assigned to a species-level grouping according to 97 or 98% sequence similarities for Basidiomycota and Ascomycota, respectively (Nilsson et al. 2008), using the furthest neighbor algorithm in DOTUR (Schloss and Handelsman 2005). Representative DNA sequences were later re-checked against the GenBank and UNITE sequence databases to assign a taxonomic name to each group. Individual sequences were deposited in GenBank (KX852463, KC791018–KC791119, Table S1).

### Statistical analysis

Following the rainfall event on 30 and 31 July, ECM fungal taxa were classified into rare and common species based on their frequency of appearance at the five sampling times of six quadrats at each NR and WA treatment. For both the NR and WA treatments, ECM fungal species that were found only once or twice (frequency of appearance (%) < 40%) in any single sample at each sampling date were defined as rare species, whereas ECM fungal species that were found four or more times (Frequency of appearance (%) > 80%) were defined as common species. Separate rarefaction curves were made for number of ECM root tips of each sample from May to the beginning of August and following the rainfall event with the vegan package (Oksanen et al. 2016) of R (version 3.1.2, R Development Core Team 2014). Fungal species diversity indices (Simpson’s diversity index and Shannon’s diversity index) were also computed with EstimateS 7.5 (Colwell 2005). The relationships among the numbers of NM or ECM root tips, the ECM colonization rate, the ECM fungal richness, the ECM fungal diversity, or the abundance of each species, genus, or family, and the volumetric soil water content (VWC) or the day of the year (DOY) from the beginning of May to August were analyzed by a regression analysis. For the data collected after the rainfall event, the relationships among the sampling dates and the numbers of NM and ECM root tips; the ECM colonization rate; the number of ECM fungal species; the ECM fungal diversity; the numbers of ECM species and ECM root tips colonized by common, rare, Ascomycota, and Basidiomycota species; and the number of ECM root tips at the species, genus, or family level were analyzed by a regression analysis. The adjusted *R*^2^ is shown in the figures. To transform the data to normal distribution, log, square root, or arcsine transformations were conducted, then numbers of NM and ECM root tips, the ECM colonization rate, the number of ECM fungal species, and the numbers of ECM species and ECM root tips colonized by Ascomycota and Basidiomycota species were analyzed by two-way repeated measures analysis of variance (ANOVA) (*P* < 0.05) to determine significant effects of the water treatment, sampling dates after the rainfall event, and the interaction. When significant effects were detected by the repeated measures ANOVA, Bonferroni multiple comparisons (*P* < 0.05) were conducted to detect the significant differences among the sampling dates after the rainfall event. The repeated measures ANOVA and regression analysis were conducted using SPSS ver. 19.0J (SPSS Japan Inc., Tokyo, Japan).

Pearson’s correlation analysis and structural equation modeling (SEM) were performed to reveal the effect of rare, common, Ascomycota, and Basidiomycota species on the total number of ECM fungal species or root tips using SPSS ver. 19.0J and the “lavaan” package (Rosseel 2012) in the R software package. The hypothesized path diagram is shown in Fig. 5a. Model fit was evaluated by comparative fit index (CFI), root mean square error of approximation (RMSEA), and standardized root mean square residual (SRMSR) values.

A non-metric multidimensional scaling (NMS) analysis was performed with the species level abundance of ECM root tips after a square root transformation. Sorensen’s (Bray–Curtis) distance was used to calculate the distance matrix among the samples. The significance of an axis was evaluated by a Monte-Carlo test. NMS was analyzed using PC-ORD 6 software (McCune and Mefford 2011, MjM Software, Gleneden Beach, OR, USA).

The most dominant species, *C. geophilum*, was omitted from the common and Ascomycota species and the analyses for ECM fungal diversity because the changes or trends in ECM fungal community and diversity were hidden by the overwhelming dominance by this fungus.

## Results

### Soil water condition

From April to the end of June, the soil water content decreased, and it remained low until the monsoon rain at the end of July (see also Allen and Kitajima 2014). After the rainfall event and WA treatment, the soil water content immediately increased and peaked 2 days later (Fig. S2c). Then, it decreased rapidly to nearly its original value after 2 and 3 weeks in the NR and WA treatments, respectively.

### Changes in NM root tips, ECM root tips, and the ECM fungal community with drying

From the automated minirhizotron images, there was a drop in number of ECM root tips from May to August (Allen and Kitajima 2014). With the small monsoonal event and the added moisture, a rapid increase in the number of new ECM tips and a corresponding increase in the relative hyphal length were observed. There was both an increase in growth and simultaneous mortality of ECM tips (Fig. 4, in Allen and Kitajima 2014).

**Fig. 1:**
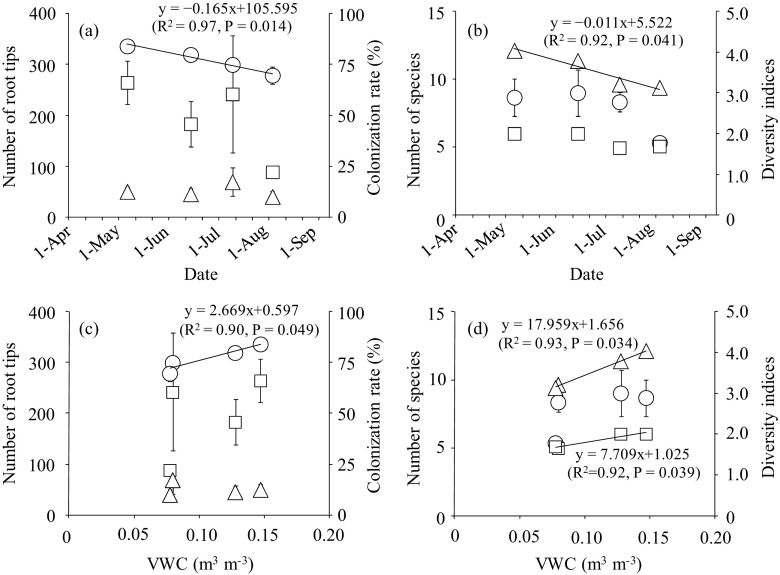
**a** Relationship between the sampling date and the number of NM roots (triangle), the number of ECM roots (square), or the ECM colonization rate (circle) before water addition. **b** The relationship between the date and the number of ECM fungal species (circle), Shannon’s diversity (square), and Simpson’s diversity (triangle) indices before water addition. **c** The relationship between the volumetric water content (VWC) and the number of NM roots (triangle), the number of ECM roots (square), and the ECM colonization rate (circle) before water addition. **d** The relationship between the VWC and the number of ECM fungal species (circle), Shannon’s diversity (square), and Simpson’s diversity (triangle) before water addition. Significant regression curves (*P* < 0.05) are shown. Bars show ± standard error (S.E.)

From the cores, the number of NM roots did not change with time, but ECM roots tended to decrease from May to August (Fig. 1a). The ECM colonization rate significantly and linearly decreased from May to August. There was a significant relationship between the ECM colonization rate and the VWC (Fig. 1c), indicating that the decrease in the ECM colonization rate from May to August was due to a decrease in soil water.

For the diversity and community analysis of ECM fungi, 2337 ECM root tips were observed under a dissecting microscope, and 183 ECM root tips were used for the molecular analysis. In total, 129 ECM root tips (approximately 70%) were successfully analyzed, and 42 ECM fungal operational taxonomic units (OTUs) were detected. The OTUs were shown in pink or yellow rows in Table S1. Average successful rates of molecular identification were 71.6, 65.5, 74.4, and 95.8% in May, June, July, and August, respectively. The ECM community comprised 16 (38%) Ascomycota and 26 (62%) Basidiomycota species, and the fraction of ECM roots colonized by Ascomycota and Basidiomycota species was 71.5 and 28.5%, respectively. The rarefaction curves for each sampling date reached a plateau, except for the July sampling (Fig. S3).

Shannon’s and Simpson’s diversity indices were lower in July and August than in May and June (Fig. 1b). A regression analysis showed that both diversity indices linearly decreased with a decreasing VWC (Fig. 1d), indicating that the decrease in the ECM fungal diversity from spring to summer was caused by soil drying.

**Fig. 2:**
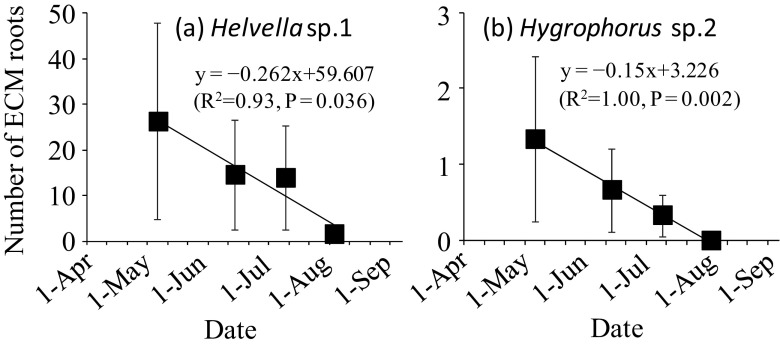
Relationship between the number of ECM root tips colonized by each ECM fungal species and the sampling date from May to August. Only the ECM fungal species with significant regression curves (*P* < 0.05) are shown. Bars show ± standard error

*C. geophilum* was the most dominant species during the study period and colonized 46 to 54% of the total mycorrhizal root tips (Fig. S4). The number of ECM roots colonized by *C. geophilum* tended to decrease with drying (data not shown), but the colonization ratio increased from May to August (Fig. S4), indicating that the drought tolerance of *C. geophilum* was higher than that of the other ECM fungal species. The number of ECM roots colonized by *Helvella* sp.1 and *Hygrophorus* sp.2 showed a significant linear decrease from May to August (Fig. 2). At genus and family level, a significant regression curve was not observed except for the genus, *Helvella*.

### Short-term changes in the numbers of ECM roots and the ECM fungal diversity after precipitation events

**Fig. 3:**
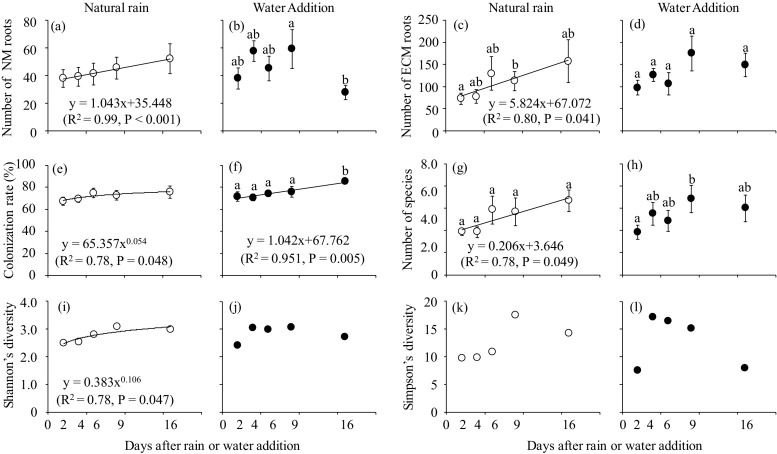
Number of NM roots in the **a** natural rain (NR) and **b** water addition (WA) treatments; the number of ECM roots in the **c** NR and **d** WA treatments; the ECM colonization rate in the **e** NR and **f** WA treatments; the number of ECM fungal species in the **g** NR and **h** WA treatments; Shannon’s diversity in the **i** NR and **j** WA treatments; and Simpson’s diversity in the **k** NR and **l** WA treatments in eight soil cores. Bars show ± S.E. Different characters indicate significant differences by a Bonferroni multiple comparison (*P* < 0.05). Significant regression curves (*P* < 0.05) are also shown

For the number of ECM roots, no significant interaction was detected between sampling date and water treatment (two-way repeated measures ANOVA, *P* > 0.05), but the effect of sampling date was significant (two-way repeated measures ANOVA, *P* < 0.001) (Table S2). The number of ECM roots in the NR treatment increased linearly, and it was significantly higher on day 9 than on day 2 (Bonferroni multiple comparison, *P* < 0.05) (Fig. 3c). In the WA treatment, it tended to be higher on days 9 and 16, compared with days 2, 4, and 6 (Fig. 3d); however, no significant differences among the sampling dates were detected (Bonferroni multiple comparison, *P* > 0.05).

The diversity and community composition of ECM fungi also changed. Eight thousand one hundred forty-one ECM root tips were observed under a dissecting microscope; 645 ECM root tips were used for the molecular analysis, and 494 ECM root tips (approximately 77%) were successfully analyzed. In total, 123 ECM fungal OTUs were detected from the analyses. The OTUs are shown in pink or blue rows in Table S1. The ECM community comprised 38 (31%) Ascomycota and 85 (69%) Basidiomycota species, and the numbers of ECM roots colonized by Ascomycota and Basidiomycota species were 52.8 and 47.2%, respectively. At the species level, the rarefaction curves for each sampling date in the NR and WA treatment reached mostly a plateau, although the curves of days 6 and 16 in the NR treatment and day 16 in the WA treatment did not and the number of ECM fungal species increased with an increasing number of ECM roots examined (Fig. S5).

The two-way repeated measured ANOVA showed that the sampling date and the interaction with water treatment had significant effects (*P* < 0.01 and *P* < 0.05, respectively) on the number of ECM fungal species (Table S2). In the NR treatment, it increased linearly (Fig. 3g). In the WA treatment, it was higher on day 9 than on day 2 (Bonferroni multiple comparison, *P* < 0.05) (Fig. 3h). In the NR treatment, the Shannon’s diversity index slightly increased from days 2 to 16 (Fig. 3i), and the Simpson’s diversity index increased on day 9 and then decreased on day 16 (Fig. 3k). In the WA treatment, both diversity indices sharply increased on day 4, remained high until day 9, and then decreased on day 16 (Fig. 3j, l).

### Short-term changes in common, rare, Ascomycota, and Basidiomycota species following precipitation events

**Fig. 4:**
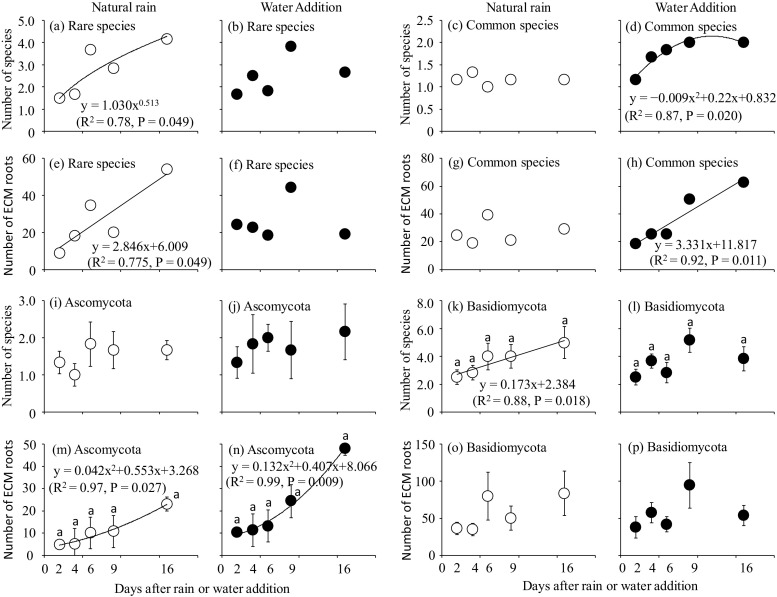
Number of rare species in the **a** NR and **b** WA treatments; the number of common species in the **c** NR and **d** WA treatments; the number of ECM root tips colonized by rare species in the **e** NR and **f** WA treatments; the number of ECM root tips colonized by common species in the **g** NR and **h** WA treatments; the number of Ascomycota species in the **i** NR and **j** WA treatments; the number of Basidiomycota species in the **k** NR and **l** WA treatments; the number of ECM root tips colonized by Ascomycota species in the **m** NR and **n** WA treatments; and the number of ECM root tips colonized by Basidiomycota species in the **o** NR and **p** WA treatments in eight soil cores. Bars show ± S.E. Different characters indicate significant differences by a Bonferroni multiple comparison (*P* < 0.05). Significant regression curves (*P* < 0.05) are shown

The numbers of ECM species and ECM root tips colonized by rare species increased as a convex curve (Fig. 4a) and a linear curve (Fig. 4e), respectively, in the NR treatment. Importantly, the numbers of ECM species and ECM root tips colonized by common species did not change in the NR treatment (Fig. 4c, g). In the WA treatment, the numbers of ECM species and ECM root tips colonized by rare species increased at day 9 and then decreased again (Fig. 4b, f). The numbers of ECM species and ECM root tips colonized by common species increased as a convex curve (Fig. 4d) and linearly (Fig. 4h), respectively.

The number of Ascomycota species did not change significantly after precipitation events in the NR and WA treatments (Table S2) (Fig. 4i, j). The number of Basidiomycota species was significantly affected by the sampling date and the interaction with water treatment (two-way repeated measures ANOVA, *P* < 0.01 and *P* < 0.05, respectively) (Table S2). The number of Basidiomycota species increased linearly in the NR treatment (Fig. 4k). In the WA treatment, it tended to increase at day 9, and then it decreased (Fig. 4l). For the number of ECM roots colonized by Ascomycota species, significant effects by the sampling date were detected by the two-way repeated measures ANOVA (*P* < 0.05) (Table S2). The number of ECM roots colonized by Ascomycota species increased as a concave curve in both NR and WA treatments (Fig. 4m, n). No significant effects were observed for the number of ECM roots colonized by Basidiomycota species by two-way repeated measures ANOVA (*P* > 0.05) (Table S2), but it tended to increase after the precipitation event in the NR treatment (Fig. 4o). In the WA treatment, the number of ECM roots colonized by Basidiomycota species tended to increase at day 9, and then it decreased on day 16 (Fig. 4p).

The number of the total ECM root tips was significantly correlated with the numbers of ECM root tips colonized by Ascomycota (*r* = 0.92, *P* < 0.05), Basidiomycota (*r* = 0.95, *P* < 0.05), and rare species (*r* = 0.95, *P* < 0.05) in the NR treatment (Table 1), and the rare species had significant correlation both with Ascomycota (*r* = 0.93, *P* < 0.05) and Basidiomycota species (*r* = 0.91, *P* < 0.05). In WA treatment, the number of the total ECM root tips was significantly correlated with the number of ECM root tips colonized by Basidiomycota species (*r* = 0.92, *P* < 0.05). The number of ECM roots colonized by Basidiomycota species had significant correlation with rare species (*r* = 0.90, *P* < 0.05), whereas Ascomycota species significantly correlated with common species (*r* = 0.94, *P* < 0.05).

**Table 1:** Pearson’s correlation coefficients between the abundance (Ab^a^) of total, rare, common, Ascomycota, and Basidiomycota species in NR (above the diagonal) and WA (below the diagonal) treatments

	Total Ab	Rare Ab	Common Ab	Asco Ab	Basidio Ab
Total Ab	–	**0.95** ^*^	0.59	**0.92** ^*^	**0.95** ^*^
Rare Ab	0.70	–	0.54	**0.93** ^*^	**0.91** ^*^
Common Ab	0.83	0.28	–	0.32	0.80
Asco Ab	0.61	− 0.02	**0.94** ^*^	–	0.81
Basidio Ab	**0.92** ^*^	**0.90** ^*^	0.55	0.25	–

Bold characters show that the correlation coefficients were significant (* < 0.05, ** < 0.01)

^a^Ab is the abbreviation of abundance (the number of ECM root tips)

**Fig. 5:**
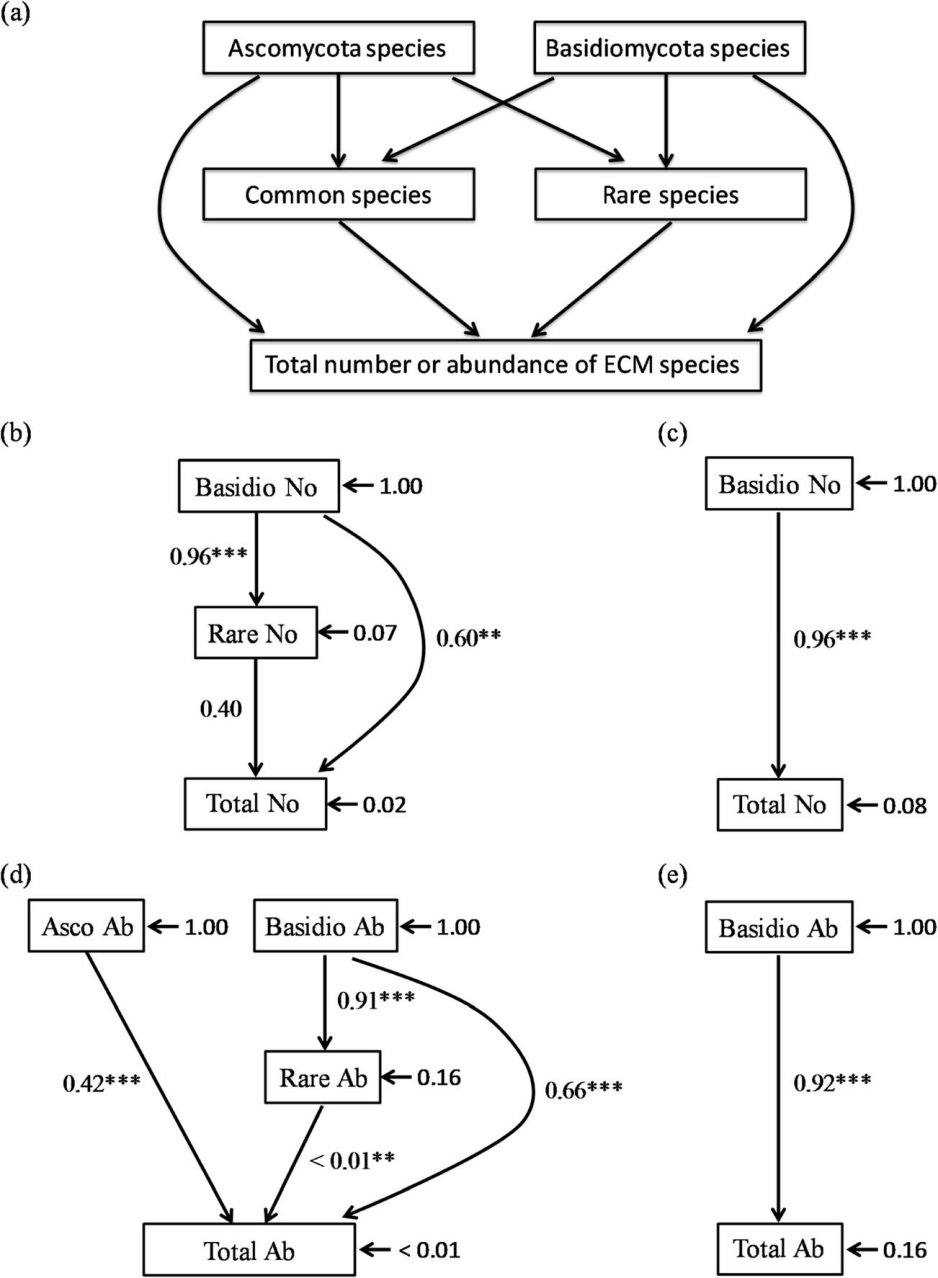
**a** The hypothesized path diagram and final structural equation model for the effects of the number of Ascomycota, Basidiomycota, rare, and common species on the total number of ECM fungal species in the **b** NR and **c** WA treatments and the effects of the number of ECM root tips (abundances) colonized by Ascomycota, Basidiomycota, rare, and common species on the total number of ECM root tips in the **d** NR and **e** WA treatment. Only significant pathways are shown (* *P* < 0.05, ** *P* < 0.01, *** *P* < 0.001)

The final SEM models are shown in Fig. 5. All of the models provided a good fit to the data (CFI = 1.00, RMSEA < 0.001, SRMSR < 0.001). The number of ECM species was significantly correlated with the numbers of rare and Basidiomycota species in the NR and WA treatments (Table 1), but the SEM model only showed a significant effect of the Basidiomycota species in both treatments (Fig. 5a, b). This was because the correlation between the number of rare and Basidiomycota species was high and it was difficult to use both variances in a model. The number of the total ECM root tips was significantly affected by the number of ECM roots colonized by Ascomycota, Basidiomycota, and rare species in the NR treatment (Fig. 5c), and it was significantly affected by the number of ECM roots colonized by Basidiomycota species in the WA treatment (Fig. 5d). These results correspond to the significance of the correlation coefficients (Table 1).

### Short-term change in the ECM fungal species composition after precipitation events

Throughout the study period, *C. geophilum* was the most dominant species, while the *Cortinarius* spp., *Helvella* sp.1, *Inocybe* spp., *Sebacina* spp., and *Tomentella* spp. were also dominant (Fig. S6). The number of ECM roots colonized by *C. geophilum* ranged from 31 to 49% in the NR treatment and from 32 to 51% in the WA treatment, and the percentage of *C. geophilum* tended to decrease with time after the precipitation events, although its abundance tended to increase in the NR treatment and it did not change in the WA treatment.

**Fig. 6:**
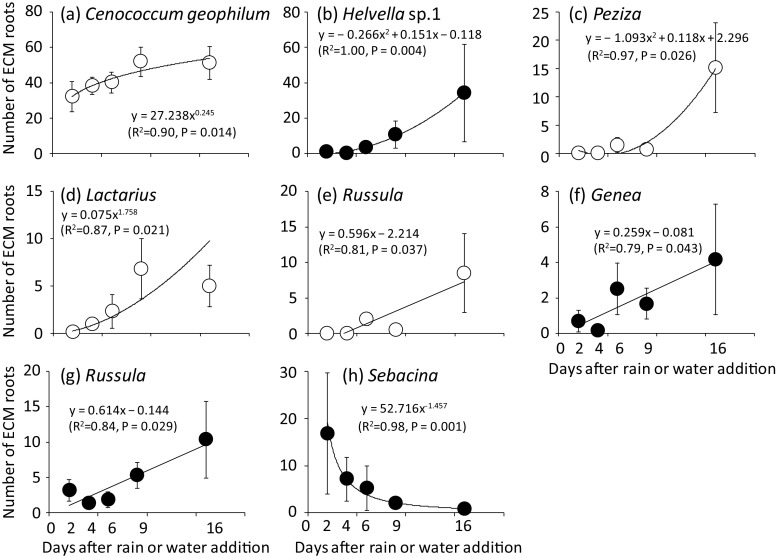
Relationship between the number of ECM root tips colonized by each ECM fungal species or genus and the date in the NR treatment (white circle) and the WA treatment (black circle). Only the ECM fungal species or genus with significant regression curve (*P* < 0.05) are shown. Bars show ± S.E

In the NR treatment, a repeated measure ANOVA showed that the number of ECM root tips colonized by *C. geophilum*, Russulaceae, and Thelephoraceae differed significantly among the sampling dates (*P* < 0.05). The results of the regression analysis showed that the number of ECM roots colonized by *C. geophilum* slightly increased after the precipitation event (Fig. 6a). At the genus level, the number of ECM root tips colonized by *Peziza* and *Lactarius* increased as a concave curve (Fig. 6c, d), and *Russula* increased linearly (Fig. 6e). In the WA treatment, *Helvella* sp.1 increased as a quadratic curve after the water supply (Fig. 6b). At the genus level, *Genea* (Fig. 6f) and *Russula* (Fig. 6g) showed linear increase in the number of ECM root tips, whereas *Sebacina* decreased as a concave curve (Fig. 6h).

The NMS analysis showed the change in the ECM community after water supply. The final stress and instability were 7.25 and < 0.000001, respectively. The cumulative coefficient of determination was 0.85, consisting of 0.58 for axis 1 and 0.26 for axis 2. A randomization test showed a significant (< 0.01) *P* value. In the NR treatment, the ECM community composition drastically changed at day 6, and it kept changing until day 16 along axis 2 (Fig. S8). In the WA treatment, the ECM community composition drastically changed at day 9 along axis 2. However, on day 16, the ECM community composition was similar to that on day 6, as it exhibited a lower *y* axis value.

## Discussion

We observed a decrease in ECM formation and diversity during dry periods, as was reported previously (Nilsen et al. 1998; Swaty et al. 2004). Importantly, neither ECM fungal hyphae nor ectomycorrhizae went to 0 (Allen and Kitajima 2014). Previous studies have shown that both ECM root tips and ECM fungal hyphae (Allen and Kitajima 2014) persist during a dry season; in part, they are sustained by hydraulic lift (Querejeta et al. 2003, 2009; Kitajima et al. 2013). As both hyphae and fine roots are still active, they are primed to activate with even minimal precipitation (Allen and Kitajima 2013, 2014).

The natural precipitation at our study site was less than 10 mm, as it was undetectable by our precipitation gauges. It did increase soil surface moisture by approximately 0.5%, and photosynthetically activate radiation values indicated cloudiness across the dates of the water inputs. Nevertheless, ECM roots (Kitajima et al. 2013) and ECM fungal hyphae (Allen and Kitajima 2014) responded to even minor water inputs, and they responded even more to the WA. However, our experimental setting does not include the control treatment that did not receive any water supply, so further research focusing on the effect of a little rain on ECM roots will be needed to exactly refer to such small water effect.

Importantly, there was measurable mortality in ECM hyphae with the precipitation (and supplemental water), but there was also a rather dramatic increase in new hyphae production. The coupled mortality and new growth provided an increased opportunity for new mycorrhiza formation. In our experimental patch, two-way repeated measures ANOVAs showed the significant interaction between the sampling dates and water treatment for the number of ECM species, but not for the number of ECM roots. However, the number of ECM roots tended to increase within 9 days following precipitation events in both the NR and WA treatments. Diversity indices increased on day 9 in the NR treatment and on day 4 in the WA treatment, indicating that the changes in the ECM abundance, diversity, and community occurred within 9 days after receiving precipitation. Burgess et al. (1996) reported *Pisolithus*–*Eucalyptus* ECM development in an in vitro system. A Hartig net began to develop after 1 day as a result of contact between fungal hyphae and lateral roots, and then the mantle thickened and the Hartig net were formed 0.3 mm behind the root apex at day 3 (Burgess et al. 1996). Martin et al. (1995) found that new infections could develop within 2 days and functioning within four. In our study, it might be possible that fast-growing ECM fungal species formed ectomycorrhizae as quickly as 4 days after receiving precipitation. Different amounts of precipitation did not change the speed of the response of individual ECM fungi, although a greater amount of precipitation resulted in more newly formed tips, with a concomitant potential for rapid increases in ECM fungal diversity.

Different amount of precipitation resulted in changes in the response of different ECM fungal groups. In the NR treatment, ECM fungal growth increased after the rainfall event. In contrast, in the WA treatment, with more water, the reaction was pulse-like, namely an increase after the WA, followed by a decrease or no change from days 9 to 16. Similar differences in the response to a greater volume of water were observed for the numbers of rare and Basidiomycota species, and the number of ECM root tips colonized by Basidiomycota species. On the other hand, the number of ECM root tips colonized by Ascomycota species increased as a concave curve in both NR and WA treatments, despite the drying of soil with time, indicating that the pulse-like change was mainly associated with Basidiomycota. This suggests that Basidiomycota species may be more sensitive to wet and dry transitions than Ascomycota species. Our results support the high drought tolerance of Ascomycota species (Gehring et al. 1998; Swaty et al. 2004; Sthultz et al. 2009). The high sensitivity of Basidiomycota species to drought might allow for the dominance of Ascomycota species in dry conditions. Importantly, drought tolerance and preferred water condition of each ECM fungal taxon differs (di Pietro et al. 2007) leading to the complex community response observed. Also, water deficit stress affects the growth rates of different ECM fungi (Coleman et al. 1989). In our study, the soil water content was higher in the WA treatment than in the NR treatment, and rapidly growing ECM fungal species with low drought tolerances appeared to have formed many new ectomycorrhizae. In the NR treatment, the volume of precipitation might have been insufficient for rapidly growing ECM fungal species with low drought tolerances, and the distribution of soil water was likely patchy in this treatment. Therefore, increases in the numbers of rare species and species with a high drought tolerance and slow growth rates, such as *C. geophilum*, might be expected.

At our research site, each ECM fungal species and genus responded differently to dry conditions and subsequent watering. Under dry conditions, the most dominant species, *C. geophilum*, tended to decrease in absolute abundance, but it increased in relative abundance. After precipitation events, the absolute abundance of *C. geophilum* increased slightly in the NR treatment, and it did not change in the WA treatment. However, its relative abundance decreased in the WA treatment. *C. geophilum* is known to be drought tolerant (Mexal and Reid 1973; Querejeta et al. 2009). Under dry conditions, the abundances of the *Helvella* sp.1 and *Hygrophorus* sp.2 decreased linearly, while the abundance of the *Helvella* sp.1 increased after precipitation events, indicating that the *Helvella* sp.1 was a mesic species. In addition, number of ECM root tips colonized by *Genea* increased in WA treatment after water supply, showing that the fungus responded rapidly to the water supply. Numbers of ECM root tips colonized by *Lactarius*, *Rusula*, and *Peziza* in NR treatment also increased after a precipitation event. These genera might respond rapidly to the small water supply, although the change also might be affected by the temporal factor such as seasonality.

Drought-adapted fungi may well provide an important carbon sequestration role in these variable environments. The turnover of ECM fungal hyphae differs among ECM fungal species (Koide and Malcolm 2009; Wilkinson et al. 2011; Fernandez and Koide 2012; Fernandez et al. 2013). *C. geophilum* appears to be decomposed slowly because of its melanin production (Koide and Malcolm 2009; Fernandez and Koide 2014), and mycorrhizae persist 4–10 times longer than other ECM fungi (Fernandez et al. 2013). In our study site, *C. geophilum* was the most dominant species, as was observed in other oak forests (Richard et al. 2005; Morris et al. 2008; Querejeta et al. 2009), and its abundance ranged from 29 to 42% and from 30 to 49% in the NR and WA treatments, respectively. The pattern of ECM root colonization by *C. geophilum* tended to be different between the NR and WA treatments. In the NR treatment, the number of ECM roots that were colonized by *C. geophilum* remained high after days 9 and 16, compared with that on day 2, whereas there was no significant difference in root colonization among the sampling dates in the WA treatment. In this Mediterranean ecosystem in CA, the numbers of ECM root tips and dominant ECM fungal species on black oak gradually increased when the amount of precipitation was less than 10 mm, but not more than 50 mm, indicating that the precipitation volume affects the number of ectomycorrhizae formed by *C. geophilum*.

The positive effects, such as nutrient uptake and growth promotion, of ECM fungi on plants also differs among ECM fungal species (Abuzinadah et al. 1986; Cairney 1999; Van der Heijden and Kuyper 2003; Taniguchi et al. 2008), and the change in the ECM fungal community composition following precipitation events might cause such functional changes in host plants. However, the relationships among the changes in the ECM fungal community and ecosystem functions, such as the decomposition of organic materials and dead mycorrhizae, and the positive effects on plants after precipitation events remain unclear.

Importantly, when assessing the overall community responses, the fungi that rapidly increased in abundance following precipitation events largely belonged to rare taxa. Common taxa were present during the drought period because they were sustained by hydraulic lift. Following precipitation events, especially in the WA treatment, infrequent, possibly dormant species rapidly responded to the moisture by colonizing the newly emerging root tips.

More work on dynamic events and thresholds are clearly needed to tease apart the activity, responses, and implications of taxonomic shifts, particularly between abundant and rare species in highly variable environments, such as regions with a Mediterranean climate. Further research from a dynamic viewpoint will elucidate the effects of global warming-mediated changes in precipitation levels on dryland ecosystems.

## Electronic supplementary material

ESM 1 (DOCX 547 kb)
