# Supplementary material for: A pulse of summer precipitation after the dry season triggers changes in ectomycorrhizal formation, diversity, and community composition in a Mediterranean forest in California, USA
Source: Mycorrhiza. 2018 Aug 13;28(7):665–77. doi: 10.1007/s00572-018-0859-3 (PMC6182365; doi:10.1007/s00572-018-0859-3)
Supplement: Supplementary file 1 — (DOCX 547 kb) [file 572_2018_859_MOESM1_ESM.docx]

**Table S1** Ectomycorrhizal (ECM) fungi detected from amplification and cloning of ECM fungal root tips in the James Reserve. Each OTU was considered as species. Consensus ECM fungal names were used at both seasonal and short-term observation.

Each color shows the appearance of each species with time (pink, both before and after rainfall; yellow, before the rainfall; blue, after the rainfall).

| Phylum | Order | Family | Tentative identification | NCBI accession no. | Length | Best BLAST match  Accession 　Query cov. (%) 　Max. ident. (%) | | |
| --- | --- | --- | --- | --- | --- | --- | --- | --- |
| Ascomycota | Helotiales |  | *Cadophora* *finlandica* sp. 1 | KC791027 | 888 | JX630499.1 | 100 | 98 |
|  | Helotiales |  | *Cadophora finlandica* sp. 2 | KC791028 | 550 | AB190393.1 | 97 | 99 |
|  | Helotiales |  | *Cadophora finlandica* sp. 3 | KC791029 | 860 | JX630499.1 | 99 | 97 |
|  | Dothideomycetes |  | *Cenococcum geophilum* | KX852463 | 487 | AY3949.1 | 100 | 99 |
|  | Eurotiales | Elaphomycetaceae | *Elaphomyces decipiens* sp. 1 | KC791023 | 644 | KR029743.1 | 100 | 98 |
|  | Eurotiales | Elaphomycetaceae | *Elaphomyces decipiens*sp.2 | KC791025 | 636 | EU846311.1 | 99 | 99 |
|  | Eurotiales | Elaphomycetaceae | *Elaphomyes* sp. 1 | KC791024 | 650 | KR029749.1 | 97 | 97 |
|  | Eurotiales | Elaphomycetaceae | *Elaphomyces* sp. 2 | KC791026 | 682 | KR029774.1 | 100 | 96 |
|  | Eurotiales |  | Eurotiales sp. 1 | KC791019 | 588 | KC007253.1 | 100 | 99 |
|  | Pezizales | Pyronemataceae | *Genabea* sp.1 | KC791053 | 745 | DQ206864.1 | 70 | 95 |
|  | Pezizales | Pyronemataceae | Genabea sp.2 | KC791054 | 633 | DQ206864.1 | 94 | 92 |
|  | Pezizales | Pyronemataceae | Genabea sp.3 | KC791055 | 622 | DQ206864.1 | 94 | 91 |
|  | Pezizales | Pyronemataceae | *Genea gardneri* | KC791050 | 690 | DQ206860.2 | 100 | 99 |
|  | Pezizales | Pyronemataceae | *Genea* sp. 1 | KC791045 | 693 | DQ206861.1 | 94 | 98 |
|  | Pezizales | Pyronemataceae | *Genea* sp. 2 | KC791046 | 748 | JN022508.1 | 91 | 90 |
|  | Pezizales | Pyronemataceae | *Genea* sp. 3 | KC791047 | 718 | JN022508.1 | 91 | 97 |
|  | Pezizales | Pyronemataceae | *Genea* sp. 4 | KC791048 | 693 | DQ218282.1 | 98 | 96 |
|  | Pezizales | *Pyronemataceae* | *Genea* sp. 5 | KC791049 | 694 | DQ206860.2 | 100 | 96 |
|  | Pezizales | Pyronemataceae | *Gilkeya compacta* | KC791051 | 580 | DQ206862.1 | 93 | 99 |
|  | Helotiales |  | Helotiales sp. 1 | KC791018 | 768 | FN185979.1 | 70 | 85 |
|  | Helotiales |  | Helotiales sp. 2 | KC791031 | 560 | KF359568.1 | 100 | 96 |
|  | Helotiales |  | Helotiales sp. 3 | KC791032 | 556 | NR121307.1 | 98 | 97 |
|  | Helotiales |  | Helotiales sp. 4 | KC791033 | 549 | NR121307.1 | 98 | 97 |
|  | Helotiales |  | Helotiales sp. 5 | KC791034 | 535 | KF359568.1 | 100 | 99 |
|  | Pezizales | Helvellaceae | Helvellaceae sp. 1 | KC791036 | 754 | JF506759.1 | 100 | 100 |
|  | Pezizales | Helvellaceae | Helvellaceae sp. 2 | KC791037 | 816 | JX462562.1 | 94 | 84 |
|  | Pezizales | Helvellaceae | *Helvella* sp. 1 | KC791035 | 699 | KC122832.1 | 98 | 100 |
|  | Helotiales |  | *Meliniomyces* sp. 1 | KC791030 | 554 | AB543057.1 | 100 | 97 |
|  | Pezizales | Pezizaceae | *Peziza* sp. 1 | KC791038 | 638 | AY830851.1 | 98 | 98 |
|  | Pezizales | Pezizaceae | *Peziza* sp. 2 | KC791039 | 634 | JF908539.1 | 100 | 97 |
|  | Pleosporales |  | Pleosporales sp. 1 | KC791021 | 540 | JN859337.1 | 100 | 95 |
|  | Pezizales | Pyronemataceae | Pyronemataceae sp. 1 | KC791022 | 608 | HQ829055.1 | 100 | 86 |
|  | Pezizales | Pyronemataceae | *Trichophaea* sp. 1 | KC791042 | 575 | GQ281482.1 | 100 | 92 |
|  | Pezizales | Pyronemataceae | Pyronemataceae sp. 3 | KC791043 | 623 | JQ393151.1 | 95 | 99 |
|  | Pezizales | Pyronemataceae | Pyronemataceae sp. 4 | KC791052 | 683 | DQ974752.1 | 94 | 95 |
|  | Pezizales | Pezizaceae | *Hydnobolites* sp. 1 | KC791040 | 598 | FJ235138.1 | 93 | 97 |
|  | Pezizales | Pezizaceae | *Hydnobolites* sp. 2 | KC791041 | 584 | FJ235138.1 | 92 | 93 |
|  | Pezizales | Tuberaceae | *Tuber* sp. 1 | KC791056 | 653 | HM485363.1 | 93 | 98 |
|  | Pezizales | Tuberaceae | *Tuber* sp. 2 | KC791057 | 744 | JN022528.1 | 95 | 92 |
|  | Pezizales | Pyronemataceae | *Wilcoxina* sp. 1 | KC791044 | 591 | AF266708.1 | 100 | 99 |
|  |  |  |  |  |  |  |  |  |
| Basidiomycota |  |  | Agaricomycetes sp. 1 | KC791140 | 617 | AB831858.1 | 68 | 86 |
|  | Agaricales | Amanitaceae | *Amanita* sp. 1 | KC791058 | 629 | AB080784.1 | 100 | 100 |
|  | Atheliales | Atheliaceae | Atheliaceae sp. 1 | KC791144 | 591 | KP814516.1 | 100 | 87 |
|  | Atheliales | Atheliaceae | Atheliaceae sp. 2 | KC791146 | 620 | KM576314.1 | 93 | 98 |
|  | Boletales | Boletaceae | *Boletus* sp. 1 | KC791094 | 790 | KC184479.1 | 90 | 99 |
|  | Boletales | Boletaceae | *Boletus* sp. 2 | KC791095 | 701 | KC184445.1 | 90 | 99 |
|  | Cantharellales | Clavulinaceae | *Clavulina* sp. 1 | KC791096 | 427 | GQ981509.1 | 95 | 93 |
|  | Cantharellales | Clavulinaceae | *Clavulina* sp. 2 | KC791097 | 648 | KP783435.1 | 96 | 100 |
|  | Cantharellales | Clavulinaceae | *Clavulina* sp. 3 | KC791098 | 687 | EU862214.1 | 98 | 97 |
|  | Corticiales | Corticiaceae | Corticiaceae sp. 1 | KC791102 | 636 | KM576416.1 | 93 | 93 |
|  | Corticiales | Corticiaceae | Corticiaceae sp. 2 | KC791148 | 611 | KP814386.1 | 100 | 93 |
|  | Agaricales | Cortinariaceae | *Cortinarius* sp. 1 | KC791105 | 686 | KJ421029.1 | 100 | 98 |
|  | Agaricales | Cortinariaceae | *Cortinarius* sp. 2 | KC791106 | 591 | JQ724020.1 | 100 | 99 |
|  | Agaricales | Cortinariaceae | *Cortinarius* sp. 3 | KC791107 | 591 | HQ604721.1 | 100 | 99 |
|  | Agaricales | Cortinariaceae | *Cortinarius* sp. 4 | KC791108 | 605 | KP165545.1 | 100 | 95 |
|  | Agaricales | Cortinariaceae | *Cortinarius* sp. 5 | KC791109 | 595 | KX831120.1 | 100 | 97 |
|  | Agaricales | Cortinariaceae | *Cortinarius* sp. 6 | KC791110 | 598 | NR131887.1 | 100 | 100 |
|  | Agaricales | Cortinariaceae | *Cortinarius* sp. 7 | KC791111 | 604 | HQ604728.1 | 100 | 95 |
|  | Agaricales | Cortinariaceae | *Cortinarius* sp. 8 | KC791112 | 590 | HQ604719.1 | 100 | 96 |
|  | Agaricales | Cortinariaceae | *Cortinarius* sp. 9 | KC791113 | 596 | KP406558.1 | 100 | 95 |
|  | Agaricales | Cortinariaceae | *Cortinarius* sp. 10 | KC791114 | 593 | HQ604701.1 | 100 | 95 |
|  | Agaricales | Cortinariaceae | *Cortinarius* sp. 11 | KC791115 | 685 | EU057020.1 | 96 | 99 |
|  | Agaricales | Cortinariaceae | *Cortinarius* sp. 12 | KC791116 | 671 | EU056970.1 | 100 | 96 |
|  | Agaricales | Cortinariaceae | *Cortinarius* sp. 13 | KC791117 | 590 | JQ724020.1 | 99 | 99 |
|  | Cantharellales | Cantharellaceae | *Craterellus* sp. 1 | KC791143 | 973 | GU590930.1 | 97 | 87 |
|  | Agaricales | Entolomataceae | *Entoloma* sp. 1 | KC791059 | 899 | FN669198.1 | 96 | 99 |
|  | Gomphales | Gautieriaceae | *Gautieria* sp. 1 | KC791119 | 679 | AF377071.1 | 100. | 95 |
|  | Gomphales | Gautieriaceae | *Gautieria* sp. 2 | KC791120 | 700 | KC152098.1 | 100 | 98 |
|  | Gomphales | Gomphaceae | Gomphaceae sp. 1 | KC791101 | 635 | KP814533.1 | 100 | 86 |
|  | Gomphales | Gomphaceae | Gomphaceae sp. 2 | KC791118 | 680 | AF377059.1 | 100 | 92 |
|  | Agaricales | Cortinariaceae | *Hebeloma* sp. 1 | KC791103 | 694 | KX687202.1 | 100 | 99 |
|  | Agaricales | Cortinariaceae | *Hebeloma* sp. 2 | KC791104 | 695 | KT218268.1 | 100 | 99 |
|  | Agaricales | Hygrophoraceae | *Hygrophorus* sp. 1 | KC791060 | 538 | KF291216.1 | 100 | 97 |
|  | Agaricales | Hygrophoraceae | *Hygrophorus* sp. 2 | KC791061 | 534 | EF395374.1 | 91 | 95 |
|  | Agaricales | Hymenogasteraceae | *Hymenogaster* sp. 1 | KC791062 | 681 | DQ328124.1 | 97r | 99 |
|  | Hysterangiales | Hysterangiaceae | *Hysterangium* sp. 1 | KC791141 | 580 | DQ974736.1 | 88 | 93 |
|  | Hysterangiales | Hysterangiaceae | *Hysterangium* sp. 2 | KC791142 | 664 | DQ974810.1 | 94 | 98 |
|  | Agaricales | Inocybaceae | Inocybaceae sp. 1 | KC791063 | 595 | AB848499.1 | 77 | 87 |
|  | Agaricales | Inocybaceae | Inocybaceae sp. 2 | KC791064 | 687 | KX897443.1 | 99 | 86 |
|  | Agaricales | Inocybaceae | *Inocybe* sp. 1 | KC791065 | 656 | JQ801411.1 | 99 | 99 |
|  | Agaricales | Inocybaceae | Inocybaceae sp. 3 | KC791066 | 597 | AB848499.1 | 77 | 87 |
|  | Agaricales | Inocybaceae | *Inocybe* sp. 2 | KC791067 | 357 | HQ604474.1 | 100 | 99 |
|  | Agaricales | Inocybaceae | *Inocybe* sp. 3 | KC791068 | 693 | JF908235.1 | 91 | 91 |
|  | Agaricales | Inocybaceae | *Inocybe* sp. 4 | KC791069 | 668 | KF679813.1 | 100 | 94 |
|  | Agaricales | Inocybaceae | *Inocybe* sp. 5 | KC791070 | 700 | HG796992.1 | 94 | 98 |
|  | Agaricales | Inocybaceae | *Inocybe* sp. 6 | KC791071 | 675 | JQ393079.1 | 100 | 99 |
|  | Agaricales | Inocybaceae | *Inocybe* sp. 7 | KC791072 | 710 | JX630723.1 | 100 | 95 |
|  | Agaricales | Inocybaceae | *Inocybe* sp. 8 | KC791073 | 726 | HQ604778.1 | 98 | 97 |
|  | Agaricales | Inocybaceae | *Inocybe* sp. 9 | KC791074 | 667 | HQ604778.1 | 100 | 97 |
|  | Agaricales | Inocybaceae | *Inocybe* sp. 10 | KC791075 | 709 | GQ166912.1 | 94 | 95 |
|  | Russulales | Russulaceae | *Lactarius* sp. 1 | KC791080 | 737 | EU726293.1 | 93 | 99 |
|  | Russulales | Russulaceae | *Lactarius* sp. 2 | KC791081 | 889 | KJ705210.1 | 97 | 97 |
|  | Russulales | Russulaceae | *Lactarius* sp. 3 | KC791082 | 740 | EU598169.1 | 95 | 98 |
|  | Boletales | Leucogastraceae | Leucogastraceae sp. 1 | KC791076 | 576 | KT968568.1 | 98 | 89 |
|  | Boletales | Leucogastraceae | Leucogastraceae sp. 2 | KC791077 | 556 | AY621752.1 | 100 | 93 |
|  | Boletales | Leucogastraceae | Leucogastraceae sp. 3 | KC791078 | 613 | KT968583.1 | 96 | 90 |
|  | Boletales | Melanogastraceae | Melanogastraceae sp. 1 | KC791079 | 772 | KX438335.1 | 96 | 93 |
|  | Atheliales | Atheliaceae | *Piloderma* sp. 1 | KC791145 | 615 | KP814544.1 | 100 | 98 |
|  | Atheliales | Atheliaceae | *Piloderma* sp. 2 | KC791147 | 560 | JQ711862.1 | 100 | 99 |
|  | Russulales | Russulaceae | *Russula* sp. 1 | KC791083 | 669 | EU819428.1 | 100 | 99 |
|  | Russulales | Russulaceae | *Russula* sp. 2 | KC791084 | 677 | DQ422032.1 | 100 | 96 |
|  | Russulales | Russulaceae | *Russula* sp. 3 | KC791085 | 683 | KR082875.1 | 96 | 97 |
|  | Russulales | Russulaceae | *Russula* sp. 4 | KC791086 | 702 | HQ604850.1 | 100 | 98 |
|  | Russulales | Russulaceae | *Russula* sp. 5 | KC791087 | 604 | JN129407.1 | 100 | 96 |
|  | Sebacinales | Sebacinaceae | *Sebacina* sp. 1 | KC791088 | 597 | KF000461.1 | 100 | 99 |
|  | Sebacinales | Sebacinaceae | *Sebacina* sp. 2 | KC791089 | 629 | FN669246.1 | 94 | 95 |
|  | Sebacinales | Sebacinaceae | *Sebacina* sp. 3 | KC791090 | 613 | KF000411.1 | 100 | 97 |
|  | Sebacinales | Sebacinaceae | *Sebacina* sp. 4 | KC791091 | 618 | KF000421.1 | 100 | 98 |
|  | Sebacinales | Sebacinaceae | *Sebacina* sp. 5 | KC791092 | 605 | AJ534910.1 | 97 | 95 |
|  | Sebacinales | Sebacinaceae | *Sebacina* sp. 6 | KC791093 | 643 | JQ711843.1 | 100 | 97 |
|  | Corticiaceae | Sistotrema | *Systotrema* sp. 1 | KC791099 | 618 | KP814242.1 | 100 | 99 |
|  | Corticiaceae | Sistotrema | *Systotrema* sp. 2 | KC791100 | 623 | KP814242.1 | 100 | 92 |
|  | Thelephorales | Thelephoraceae | Thelephoraceae sp. 1 | KC791130 | 671 | JF273548.1 | 97 | 93 |
|  | Thelephorales | Thelephoraceae | *Tomentella* sp. 1 | KC791121 | 667 | AF430259.1 | 97 | 96 |
|  | Thelephorales | Thelephoraceae | *Tomentella* sp. 2 | KC791122 | 665 | JX630675.1 | 100 | 97 |
|  | Thelephorales | Thelephoraceae | *Tomentella* sp. 3 | KC791123 | 665 | KC152246.1 | 100 | 98 |
|  | Thelephorales | Thelephoraceae | *Tomentella* sp. 4 | KC791124 | 664 | EU444541.1 | 95 | 99 |
|  | Thelephorales | Thelephoraceae | *Tomentella* sp. 5 | KC791125 | 664 | HQ215815.1 | 89 | 99 |
|  | Thelephorales | Thelephoraceae | *Tomentella* sp. 6 | KC791126 | 665 | U92537.1 | 94 | 99 |
|  | Thelephorales | Thelephoraceae | *Tomentella* sp. 7 | KC791127 | 674 | U83471.1 | 99 | 94 |
|  | Thelephorales | Thelephoraceae | *Tomentella* sp. 8 | KC791128 | 615 | HQ215824.1 | 94 | 97 |
|  | Thelephorales | Thelephoraceae | *Tomentella* sp. 9 | KC791129 | 664 | EF644116.1 | 94 | 94 |
|  | Thelephorales | Thelephoraceae | *Tomentella* sp. 10 | KC791131 | 663 | U83473.1 | 99 | 98 |
|  | Thelephorales | Thelephoraceae | *Tomentella* sp. 11 | KC791132 | 664 | JQ272375.1 | 100 | 96 |
|  | Thelephorales | Thelephoraceae | *Tomentella* sp. 12 | KC791133 | 660 | GQ979996.1 | 100 | 95 |
|  | Thelephorales | Thelephoraceae | *Tomentella* sp. 13 | KC791134 | 661 | EF644116.1 | 94 | 97 |
|  | Thelephorales | Thelephoraceae | *Tomentella* sp. 14 | KC791135 | 666 | U83469.1 | 100 | 96 |
|  | Thelephorales | Thelephoraceae | *Tomentella* sp. 15 | KC791136 | 663 | AB605659.1 | 100 | 95 |
|  | Agaricales | Tricholomataceae | *Tricholoma* sp. 1 | KC791137 | 458 | JN389314.1 | 97 | 99 |
|  | Agaricales | Tricholomataceae | *Tricholoma* sp. 2 | KC791138 | 689 | AF377200.1 | 95 | 99 |
|  | Agaricales | Tricholomataceae | *Tricholoma* sp. 3 | KC791139 | 686 | FJ845442.1 | 99 | 98 |

**Table S2** F and P value of two-way repeated measures analysis of variances (ANOVAs) on number of NM roots, number of ECM roots, colonization rate, number of ECM species, number of Ascomycota ECM roots, number of Basidiomycota ECM roots, number of Ascomycota species, number of Basidiomycota species of water experiment. Data were analyzed with the two-way repeated measures ANOVAs with sampling date (Day), water treatment (Water) as main factors, including interactions between sampling date and water treatment (Day x Water).

Significant effects (*P* < 0.05) are shown in bold.

|  | No. of NM roots | | No. of ECM roots | | Colonization rate | | No. of ECM species | | No. of Ascomycota ECM roots | | No. of Basidiomycota ECM roots | | No. of Ascomycota species | | No. of Basidiomycota species | |
| --- | --- | --- | --- | --- | --- | --- | --- | --- | --- | --- | --- | --- | --- | --- | --- | --- |
|  | *F* | *P* | *F* | *P* | *F* | *P* | *F* | *P* | *F* | *P* | *F* | *P* | *F* | *P* | *F* | *P* |
| Day | 1.45 | 0.237 | 6.95 | **<0.001** | 4.66 | **0.004** | 5.36 | **0.001** | 3.33 | **0.019** | 1.78 | 0.152 | 1.02 | 0.408 | 4.45 | **0.005** |
| Water | 0.01 | 0.907 | 0.5 | 0.495 | 0.69 | 0.427 | 0.4 | 0.539 | 2.45 | 0.148 | 0.02 | 0.896 | 0.76 | 0.405 | 0.26 | 0.625 |
| Day x Water | 2.83 | **0.037** | 1.88 | 0.132 | 0.94 | 0.452 | 3.59 | **0.014** | 0.3 | 0.88 | 2.28 | 0.078 | 0.22 | 0.926 | 3.51 | **0.015** |


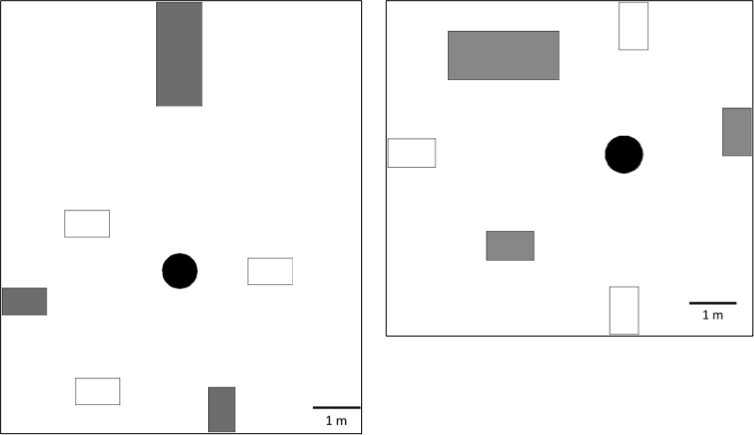


Fig. S1 Location of two black oak trees and quadrats to examine short-term responses of ECM fungal community to precipitation. Each tree was 32.0 m away each other.

Black circle: black oak; white square: natural rainfall treatment; grey square: water addition treatment; large grey square: quadrat with a soil ecosystem observatory.

**Fig. S2** (a) Daily means of soil temperature (solid line) and VWC (dotted line) in the NR treatment from April to September in 2011. Detail information on (b) soil temperature, (c) VWC, (d) soil CO_2_ concentration, and (e) soil respiration in the NR (solid line) and WA (dotted line) treatments. The vertical line shows the date when it rained and the experiment concerning the short-term effects of rain on ECM fungi started.

**Fig. S3** Rarefaction curves of ECM fungi before water addition. Each line shows the sampling times in May, June, July, and August. The rarefaction curve was composed of 42 OTUs shown in Table S1.

**Fig. S4** Relative abundance of ECM fungi at (a) species, (b) genus, and (c) family level from May to August. ECM species, genus or, family with more than 5% abundance at a sampling time are shown. *Cenococcum geophilum* is not included in the figure at genus and family level.

**Fig. S5** Rarefaction curve of the (a) natural rain (NR) and (b) water addition (WA) treatments. Each line shows the sampling date (2, 4, 6, 9, and 16 d after rain or water addition).

**Fig. S6**  Relative abundance of ECM fungi at species, genus, and family level on each sampling date in the (a, c, e) natural rain and (b, d, f) water addition treatments. ECM species, genus or, family with more than 5% abundance at a sampling time are shown. *Cenococcum geophilum* is not included in the figure at genus and family level.

**Fig. S7** Relationship between the abundance of each ECM family and the date in the (a – i) NR treatment and the (j *-* r) WA treatment. Significant regression curves (*P* < 0.05) are shown. Bars show ± S.E.

**Fig. S8** NMS analysis of the ECM fungal community at the species level in the NR and WA treatments. White and black symbols show the ECM fungal communities in the NR and WA treatments, respectively. Each symbol shows the number of days after precipitation events (circle, 2 d; triangle, 4 d; square, 6 d; diamond, 9 d; inverted triangle, 16 d).
